# Prescription Department

**Published:** 1893-02

**Authors:** 


### LA GRIPPE OR INFLUENZA COLDS.

Few remedies are more reliable and act better as a preventive, or lessen the distressing symptoms of an influenza cold, than the following mixture, says the *British and Colonial Druggist:*

℞. Sodii salicylat, ʒ jss.
Liq. ammon. acet., ℥ ij.
Aq. camph., ad ℥ vj.
Misce. Capt.: ℥ ss. every third hour.
Sig. If this be taken every two or three hours when the first symptoms of cold come on, it will usually ward off the attack.

### LA GRIPPE.

*By Prof. Dayton Parker*, Detroit, Mich.

℞. Sulphate of morphine, gr. j.
Ext. colocynth, gr. iij.
Phenacetin, gr. xxx.
M. Div. in 15 capsules.
Sig. Take once in three hours.

### SICK STOMACH.

℞. Aquæ calcis, ℥ ij.
Creasoti (Beechwood), gtt. iv.
M. Sig. Teaspoonful every fifteen minutes till stomach is quiet.

### TOPICAL APPLICATIONS IN DIPHTHERIA.

*By Geo. W. Peck, M. D.*, Omaha, Neb.

It can be employed in all stages of the disease, without danger to the patient. It should be applied often and thoroughly, by means of a brush or cotton swab, until the diseased membrane entirely disappears. In connection with this remedy I give as much brandy as the patient will bear, and such other remedies as may be indicated.

℞. Acidi borici, ʒ j.

Acidi lactici, f ʒ j.

Glycerini, f ℥ jss.

Aquæ dest., f ʒ ijss.

Liq. ferri subsulph., f ʒ jss. M.

Sig. This solution may be used in full strength or diluted with water, as each case may require.

### ACUTE VAGINAL CATARRH.

The following injection is used in acute vaginal catarrh:

℞. Zinci sulphuric, ʒ jiss.
Aq. Fontan. dist., f ℥ xiij.
Sig. Sufficient for one injection.—*Revista Clinica e Terapeutica, 12, 1890.*

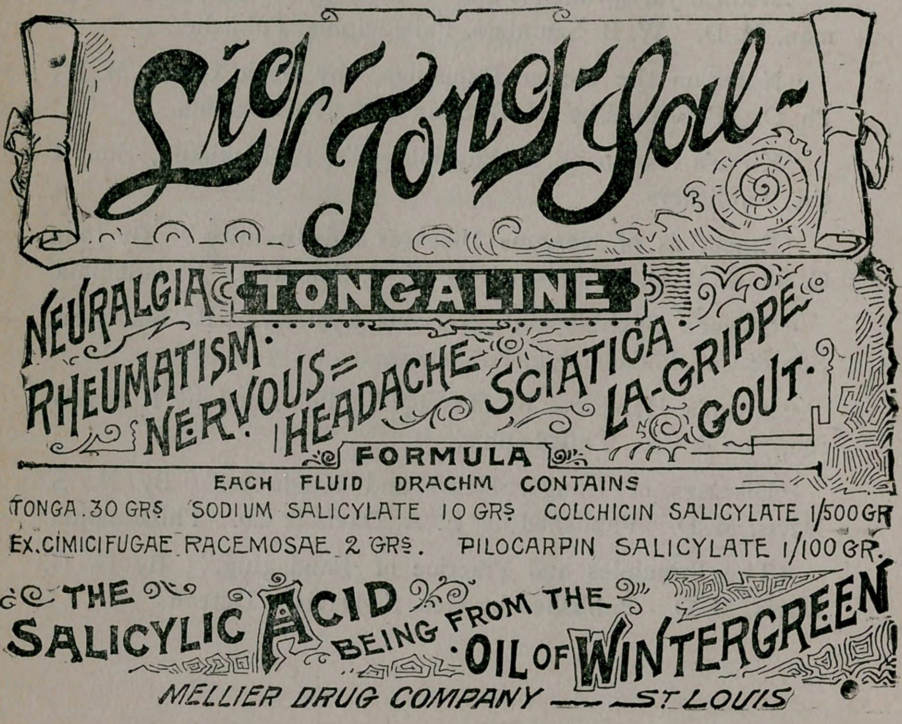

ACUTE ARTICULAR RHEUMATISM.

Dr. A. Hennig recommends the following potion in the treatment of acute articular rheumatism:

℞. Salipyrin, 6 grms.
Glycerin, 14 grms.
Raspberry-syrup, 30 grms.
Distilled water, 40 grms.

M. Sig. To be taken in the course of the afternoon, a spoonful every fifteen to twenty minutes.

CRACKED NIPPLES.

Dr. Scarff speaks highly of the following in cracked nipples:

℞. Balsam Peru, 1½ drachms.

Tinct. Arnicæ, 1½ drachms.

Ol. Amydg. Dulc, 1 ounce.

Aq. Calcis, 4 drachms.

M. Sig. Apply topically after each time the child suckles.

CRAVING FOR ALCOHOL.

The following may be given with benefit to allay the craving for alcohol, and to some extent takes its place:

℞. Spt. Ammon. Aromat. 2 ounces.

Tinct. Cinchonæ, 2½ ounces.

Tinct. Capsici, 3 drachms.

M. Sig. A large teaspoonful in half a tumblerful of effervescing potash water every hour.

CHRONIC INFLAMMATION OF TRHOAT.

*By Prof. Hiram Christopher*, St. Joseph, Mo.

℞. Vaseline, ℥j.

Eucalyptol, gtt v.

M. Spray.

ANODYNE SALVE FOR HEMORRHOIDS.

℞. Cocain Hydrochlor, 18 grains.
Morphinæ Sulph, 4½ grains.
Atropinæ Sulp, 3 grains.
Tannin, 3½ grains.
Petrolati, 5 drachms.

M. ft. ungt. Sig. Apply after each evacuation.

CHAPPED HANDS.

The *Times and Register* gives this formula for chapped hands:

℞. Menthol, 12 grammes.
Salol, 30 grammes.
Ol olive, 30 grammes.
Lanolin, 1½ oz. M.

Sig. Apply twice daily.

DIPHTHERIA.

*By Prof. J. Lewis Smith*, England.

℞. Acidi carbolici.
Ol. Eucalypti, aa f℥j.
Ol. terebinthinæ, f℥viij. M.

Sig. One ounce in a quart of water to be kept simmering on a stove in the sick room.

